# Surgical treatment of urachal remnants in an adult population—a single-centre experience

**DOI:** 10.1007/s11845-023-03339-0

**Published:** 2023-03-20

**Authors:** Paul C. Ryan, Caroline Kelly, Irfan Afridi, Aisling Fawaz, Mohammed Aboelmagd, Ivor M. Cullen, John P. Keane, Padraig J. Daly

**Affiliations:** Department of Urology, University Hospital Waterford, Dunmore Road, Co., Waterford, X91 ER8E Ireland

**Keywords:** Partial cystectomy, Transurethral resection of bladder tumour, Urachal adenocarcinoma, Urachal remnant

## Abstract

**Background:**

Urachal remnants are a rare congenital defect resulting from failure of obliteration of a fibrous tube that connects the umbilicus to the bladder dome during embryological development. Oftentimes a urachal remnant will go undiagnosed, but occasionally a patient may present with a variety of symptoms, ultimately leading to the identification of the remnant. Given its rarity, there is very limited literature available on the management of symptomatic urachal remnants, especially in adults. Surgical resection has been the first-line management of urachal remnants for years, especially given the risk of the development of urachal adenocarcinoma secondary to recurrent infection, persistent irritation, and urinary stasis associated with some urachal remnants.

**Aim:**

We present our experience in the management of symptomatic urachal remnants in adults at our institute and perform a brief literature review of the same.

**Methods:**

A retrospective review of all cases who underwent surgical management of symptomatic urachal remnants between December 2015 and January 2022 was performed. Seven cases of urachal remnant excision in total were identified over the time period. Patient characteristics and perioperative parameters were analysed. Post-operative complications were measured in accordance with the Clavien-Dindo grading system.

**Result:**

In total, 7 cases of urachal remnants were treated at our institute over the study period. Four patients were treated with a TURBT and 3 patients were treated with a laparoscopic partial cystectomy. There were no intraoperative complications and one post-operative complication requiring readmission for intravenous antibiotics. There was one mortality but this was not as a direct result of the operative procedure. Mean length of stay was 1.71 days. Two of patients had histologically confirmed urachal adenocarcinoma and the remaining five patients had benign histology. Each patient was seen in the outpatients department 6 weeks post-operatively for clinical review and review of histology. No further follow-up was required for the patients with benign histology given resolution of symptoms and follow-up for the malignant histology was arranged appropriately following MDM.

**Conclusion:**

There is a paucity of data available on the management of urachal remnants in the adult population; however, an endoscopic or laparoscopic approach is a safe and effective method of excising symptomatic urachal remnants.

## Introduction

The urachus is a normal embryonic structure that originates from the allantois and the cloaca and connects the bladder dome to the umbilicus. The urachus typically obliterates during gestational development and becomes the median umbilical ligament [1]. Occasionally, there is a failure of this obliterative process, which can lead to a number of different urachal anomalies [2]. Failure in obliteration is a rare occurrence and affects approximately 0.063% of adults [3].

The identification of urachal remnants has increased with the improvements in cross-sectional imaging; however, there is no definitive consensus on the management of urachal remnants with surgical resection often being accepted as the best management approach, given the association of urachal remnants with carcinoma of the urachus secondary to recurrent infection, inflammation, and exposure to urinary stasis [2]

We present 7 cases of urachal remnants, diagnosed and then treated surgically in a university teaching hospital between 2015 and 2022. Although the sample size is small, we believe it to be the first study evaluating surgical management of urachal remnants in an adult population in our country.

## Methods

A retrospective review of the records of patients who underwent surgical excision of urachal remnants in our institution between December 2015 and January 2022 (73 months) was performed. Cases were identified from a hospital laboratory database, and a patient chart review was performed for each individual case. Imaging was acquired from the National Integrated Medical Imaging System (NIMIS), and all patients were discussed at a multi-disciplinary meeting (MDM) preoperatively, where the decision was made on operative technique.

Seven cases of urachal remnant excision in total were identified over the time period. Patient characteristics (age, gender, symptoms, and diagnosis) perioperative parameters (surgical technique, catheter duration, length of stay, intraoperative complications, postoperative complications, and final histology) were analysed. Postoperative complications were measured in accordance with the Clavien-Dindo grading system [4].

### Surgical technique

Surgical excision is via cystoscopy and transurethral resection of bladder tumour (TURBT) or through a laparoscopic approach with partial cystectomy performed by two of three consultant surgeons.

TURBT is performed in the standard fashion with the patient placed in lithotomy position under general anaesthetic (GA) and antimicrobial cover. Following careful inspection of the urethral and bladder mucosa with a 30° and then a 70° lens, a 21ch cystoscope is replaced with a 26ch bipolar resectoscope. Complete resection of the urachal anomaly is performed, with haemostasis ensured prior to a decision being made on urethral catheter insertion (and duration of same) by the operating surgeon.

A laparoscopic partial cystectomy is performed by two of the three surgeons at our centre. All cases are performed under GA and with antimicrobial cover based on previous sensitivities cultured on midstream urine (MSU) samples sent on each patient preoperatively. One surgeon performs a Collins Knife™ incision of the perimeter of the urachal anomaly with a 26fr resectoscope using the TURBT technique prior to commencement of the laparoscopic partial cystectomy. This is to aid with the dissection of the margins of the tumour during the laparoscopic part of the procedure. A urethral catheter is routinely placed prior to laparoscopic excision so as to decompress the bladder during the initial dissection and to then allow retrograde filling and subsequent distension of the bladder prior to final excision of the urachus.

Briefly, the patient is placed in supine position with the surgeon and the surgical assistant positioned to the right of the patient. Hasson’s open technique is employed to insert the first 12-mm trocar just medial to the umbilicus. Pneumoperitoneum is achieved with insufflation of CO_2_ to a pressure of 14 mmHg. A 10-mm, 30° laparoscopic camera is placed through this port and two 5-mm working ports are placed under direct vision, one just medial to the anterior axillary line close to the anterior superior iliac spine (ASIS) on the right side and the second in the right upper quadrant, 2 cm below the costal margin at the position of the anterior axillary line.

A wide local excision of the urachal remnant is achieved using a ratcheted grasper to hold the remnant and an *ethicon harmonic ACE* + *7 shears* for haemostatic excision. Dissection of the urachal remnant is performed cranially from the transversalis fascia to the umbilicus and caudally to the space of Retzius at the point of insertion of the urachal remnant into the bladder dome. With the laparoscopic approach, avoidance of concurrent umbilectomy was deemed appropriate in the case with confirmed adenocarcinoma as clear margins could be ensured with excision of the remnant close to the transversalis fascia. In the more traditional, open approach, a partial cystectomy for urachal adenocarcinoma often includes an umbilectomy and subsequent reconstruction of the abdominal wall, as this approach taken with a midline laparotomy makes this technically easier to perform. The margins of the urachal remnant are completely excised from the bladder dome with the laparoscopic approach and then transected near the umbilicus. The cystotomy is then closed in a watertight fashion using a running 0 V-Lok suture. A drain is then placed in close proximity to the cystotomy site. The specimen is retrieved with a laparoscopic entrapment bag through the midline assistant port.

Each patient underwent an enhanced recovery protocol. The drain was removed day 2 post-op with urethral catheter typically removed day 10–14 following fluoroscopic-guided cystography.

Each patient was reviewed in the general outpatients department 6 weeks postoperatively where a clinical assessment was performed, histology was discussed, and a clinical follow-up regime was made where required.

## Results

Seven cases of urachal remnant excision were performed over the study period of 73 months. Three (42.9%) for urachal cysts, two (28.57%) were for anomalies suspicious for adenocarcinoma (one confirmed on biopsy) on cross-sectional imaging, and two (28.57%) were for a persistent patent urachus.

The mean age undergoing treatment was 34.51 years (range, 16.42–69.75). There were four (57.1%) male patients and three (42.9%) female patients. Four patients presented with abdominal discomfort prior to being diagnosed with a urachal anomaly on cross-sectional imaging (CT abdomen-pelvis), one (14.3%) was found to have a urachal anomaly incidentally on positron emission tomography (PET) imaging performed as part of surveillance for a previous malignant melanoma of skin primary, one patient presented with recurrent urinary tract infections (UTIs), and one with new onset nocturnal enuresis on a background of Di George syndrome. Each patient had dedicated computed tomography imaging prior to surgical intervention (Table 1).

**Table 1:** Perioperative data

Surgical approach, number (%)	
TURBT	4 (57.1%)
Laparoscopic partial cystectomy	3 (42.9%)
Histology, number (%)	
Benign	5 (71.4%)
Malignant	2 (18.6%)
Intraoperative complications	0 (0%)
Post-operative complications	1 (14.3%)
Mean length of stay, days (range)	1.71 (0–4)
30-day readmission	1 (14.3%)

Four patients were treated with a TURBT and three with a laparoscopic partial cystectomy. Urethral catheters were left in situ postoperatively in two (50%) of the four patients who underwent TURBT. Mean catheter duration for the remaining two patients was 12 days (range, 10–14). One of the TURBT groups showed evidence of diffuse metastatic disease so underwent a diagnostic resection of the urachal remnant to confirm adenocarcinoma prior to commencing systemic treatment. This patient passed away 8 months following their TURBT secondary to the malignancy.

Mean catheter duration for the three patients who underwent laparoscopic partial cystectomy was 15.33 days (range, 10–21), and each had a drain placed, which was removed day 2 postoperatively.

There were no intraoperative complications and one postoperative complication (Clavien-Dindo grade II) requiring readmission for intravenous antibiotics for a urinary tract infection. This was the only 30-day readmission. There was one mortality outlined above that was associated with consequences of the advanced disease on presentation. Mean length-of-stay was 1.71 days (range, 0–4).

Two (28.57%) of patients had histologically confirmed urachal adenocarcinoma, and the remaining five (71.43%) of patients had benign histology. Each patient was seen in the outpatient department 6 weeks postoperatively for clinical review and review of histology. No further follow-up was required for the patients with benign histology, given resolution of symptoms and follow-up for the malignant histology was arranged appropriately following consultation at a MDM.

## Discussion

The diagnosis of urachal remnants has increased over time with both improvements in and increasing use of cross-sectional imaging^2^. The vast majority of urachal remnant anomalies are benign with a reported incidence of malignancy associated with the urachus in < 1% of urachal anomalies [5, 6].

**Fig. 1:**
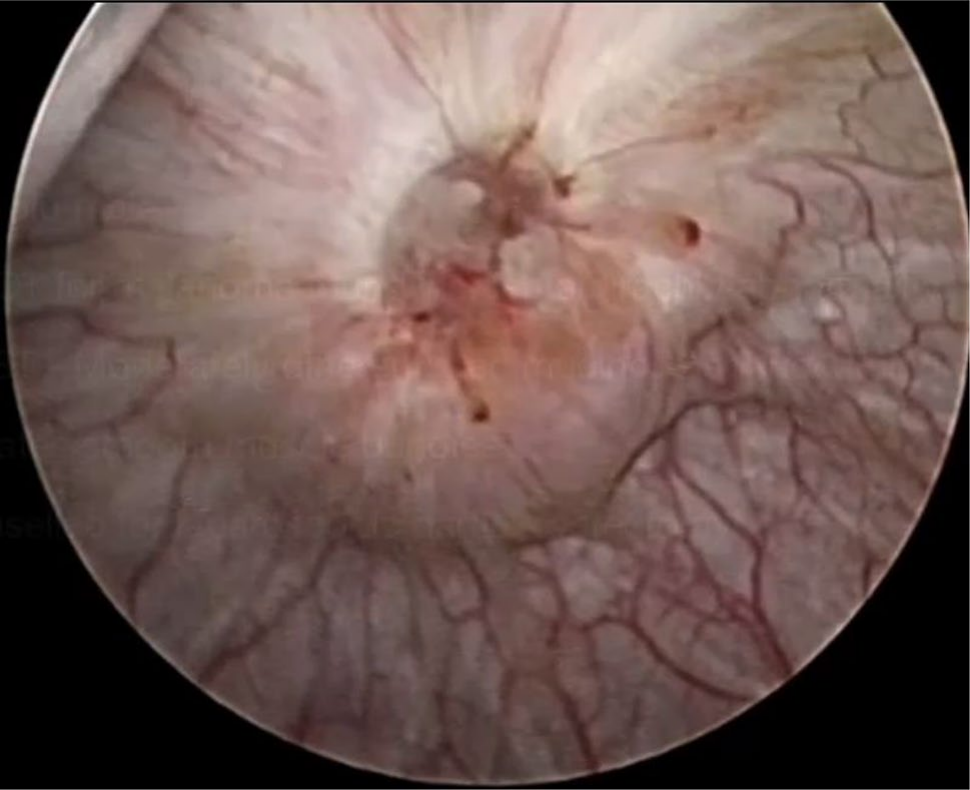
Cystoscopic view of biopsy proven urachal adenocarcinoma

In our series, the decision to treat the symptomatic urachal anomalies was made at a MDM. At this meeting, each case was discussed individually with the type of anomaly, patient symptoms, and appropriate surgical treatment investigated. A laparoscopic partial cystectomy was the treatment of choice for 3 cases. Two of these cases were for an infected patent urachus, and the third was for a biopsy-proven mucinous adenocarcinoma without metastases evident on cross-sectional imaging (Fig. 1). Three urachal cysts were treated with a TURBT, and the fourth TURBT was performed for a bladder tumour suspicious for metastatic disease, from which histology again proved to be mucinous adenocarcinoma of urachal origin. The latter passed away 8 months following the TURBT due to complications of their metastatic disease and unrelated to the surgery performed.

The incidence of urachal pathologies in adulthood is rare, as the obliteration of the urachus usually occurs during childhood [7]. There is also a paucity of data supporting the management of urachal remnants in adulthood, with most case series involving the paediatric population [2, 8]. The literature supporting the management of urachal adenocarcinoma is, therefore, even more sparce, emphasised, in part, by a recent consensus statement published by the Canadian Urology Association in which 300 publications were analysed, none of which included a prospective study or a phase 3 study [9].

The most common benign complication of urachal anomalies is infection [10]. Infective presentations are similar to that of an infection at any other site on the urinary tract but can also include erythematous skin change around the umbilicus, umbilical discharge, and/or a suprapubic mass, which may represent an inflamed umbilical cyst. As most urachal anomalies are presumed benign, there is little data available in the literature describing the appropriate level of benign management. The majority of the literature investigates the management of benign urachal anomalies in the paediatric population and does not include the adult population [2, 11–13]. The operative technique of choice in the management of benign urachal anomalies in both the paediatric and the adult population tends to be the laparoscopic approach, highlighted in a number of studies [3, 8, 11–14]. The benign anomalies in our series were treated with incision and drainage (reported as TURBT) and laparoscopic partial cystectomy. In all cases, patients presented with symptomatic urachal anomalies, and their respective symptoms resolved completely following their surgery.

In a study of similar size, Jeong et al. treated six cases of complicated urachal remnants laparoscopically [15]. Although their outcomes were the same in that each patient had their symptoms successfully treated, the perioperative outcomes with regard to catheter duration and length of hospital stay between the laparoscopic approach and TURBT approach vary (Table 2). Within our own series, we can acknowledge that the laparoscopic approach requires an overnight hospital admission in all cases, whereas two of the TURBT cases were performed as day cases. In both of these cases, the patients were discharged with no catheter left in situ. The mean length of catheter duration for the remaining two TURBT cases was 12 days, secondary to a deep tissue resection and caution raised by the operating surgery over the possibility of a perforation at the time of surgery. Mean catheter duration was 15.33 days for the laparoscopic approach in our series. In the aforementioned series reported by Jeong et al., 6 of their 7 cases involved urachal cysts, all of which were treated laparoscopically, with hospital stay ranging from 7 to 22 days but a mean catheter duration of 7.2 days. A urachal cyst may not necessarily be accessible from the transurethral approach; however, given all three cysts in our series were treated successfully by TURBT, it raises the question as to whether the laparoscopic technique is necessary to treat this type of urachal anomaly.

**Table 2:** Patient demographics and symptoms

Patient number	7
Mean age, year (range)	34.51 years (range 16.42–69.75)
Male gender, number (%)	4 (57.1%)
Symptoms, number (%)	
Abdominal discomfort	4 (57.1%)
Recurrent UTI’s	1 (14.3%)
Nocturnal enuresis	1 (14.3%)
Asymptomatic/incidental finding	1 (14.3%)

Approximately 0.5% of bladder cancers are neoplasms originating from the urachus [16]. The rarity of this cancer has meant that its management provides somewhat of a conundrum for the treating physicians as the literature available is limited. Histologically, urachal malignancy is most frequently an adenocarcinoma, and patients typically present with advanced disease resulting in a poor prognosis [17]. The management of urachal malignancies has long been surgical removal of whole, or part of the bladder; however, the long-term survival does not vary depending on which surgical approach is taken [18]. In a study performed by Ashley et al., they reviewed 130 patients who were diagnosed with urachal masses at their institute between 1951 and 2004. Sixty-six of the patients included in the study had a urachal malignancy. Interestingly, they did not find any long-term survival difference between partial vs. radical cystectomy for this disease, nor did they find that adjuvant treatment with chemotherapeutic agents was associated with a significant cancer-specific survival [18].

The long-term survival of a patient diagnosed with urachal carcinoma is not positive. In their review of the 66 patients with urachal cancer, Ashley et al. found that those initially receiving treatment for local disease had a non-metastatic recurrence in 4.8 months and only 4 of the 66 patients were found to be disease free at 14.8 years, all of whom had undergone salvage surgery for local recurrence at some point [18].

Although long-term results are similar when comparing partial with radical cystectomy, the majority of papers support a concurrent excision of the umbilicus and umbilicoplasty at the time of surgery [9, 13, 18]. At our institute, we did not surgically excise the umbilicus as two of the three cases undergoing partial cystectomy were for benign disease, and in the one case for biopsy-proven malignancy, preoperative imaging suggested local disease. A decision was made preoperatively at the MDM to avoid umbilectomy in our one case of laparoscopic partial cystectomy for adenocarcinoma, given evidence on the PET-CT that the disease did not extent beyond the perivesical fat; thus, it was deemed that complete excision of the urachus to the transversalis fascia and preserving the abdominal musculature and overlying umbilicus would allow for an adequate oncological result. The histological margins were clear of disease and with the evidence presented by Ashley et al.; it is unclear whether excision of the umbilicus would result in cancer-free survival benefit for the patient.

## Conclusion

Urachal anomalies are a rare entity that almost never require surgical intervention. When intervening, the approach taken is dependent on the underlying anomaly. The transurethral approach is safe and appropriate in treating patients with symptomatic, benign urachal cysts, whereas a laparoscopic partial cystectomy can be deployed safely to treat symptomatic, benign anomalies such as a persistent urachus.

Urachal malignancy is very rare. The prognosis of which is poor despite surgical and systemic treatment. Surgery is still at the forefront of the treatment of this cancer, with similar long-term results for both partial and radical cystectomy. Prospective, collaborative studies are required in order to influence potential future guidelines.

## Data Availability

Data can be made available upon request.

## References

[1] Severson C (2011). Enhancing nurse practitioner understanding of urachal anomalies. J Am Acad Nurse Pract.

[2] Naiditch J, Radhakrishnan J, Chin A (2013). Current diagnosis and management of urachal remnants. J Pediatr Surg.

[3] Siow S, Mahendran H, Hardin M (2015). Laparoscopic management of symptomatic urachal remnants in adulthood. Asian J Surg.

[4] Katayama H, Kurokawa Y, Nakamura K (2015). Extended Clavien-Dindo classification of surgical complications: Japan Clinical Oncology Group postoperative complications criteria. Surg Today.

[5] Wein AJ, Kavoussi LP, Partin AW (2016). Campbell-Walsh urology.

[6] García LG, Ballesta B, Rodríguez Talavera J (2021). Urachal pathology: review of cases. Urol Int.

[7] Mistry K, Khatri G, Sood D (2015). Late presentation of congenital urachal sinus in a middle aged male complicated by an umbilical abscess: a case report. Egypt J Radiol Nucl Med.

[8] Aylward P, Samson K, Raynor S, Cusick R (2020). Operative management of urachal remnants: an NSQIP based study of postoperative complications. J Pediatr Surg.

[9] Hamilou Z, North S, Canil C et al (2019) Management of urachal cancer: a review by the Canadian Urological Association and Genitourinary Medical Oncologists of Canada. Can Urol Assoc J 14(2) 10.5489/cuaj.5946 PMC7053367 31348743

[10] Yu J, Kim K, Lee H (2001). Urachal remnant diseases: spectrum of CT and US findings. Radiographics.

[11] Hashizume N, Ohtaki M, Nihei K et al (202) Laparoscopic surgery for urachal remnants in pubescent children: a case series. Surg Case Rep 6(1) 10.1186/s40792-020-00884-z PMC7266900 32488465

[12] Upadhyay V, Kukkady A (2003). Urachal remnants: an enigma. Eur J Pediatr Surg.

[13] Stopak J, Azarow K, Abdessalam S (2015). Trends in surgical management of urachal anomalies. J Pediatr Surg.

[14] Araki M, Saika T, Araki D (2012). Laparoscopic management of complicated urachal remnants in adults. World J Urol.

[15] Jeong H, Han D, Kwon W (2013). Laparoscopic management of complicated urachal remnants. Chonnam Med J.

[16] Chen D, Li Y, Yu Z (2014). Investigating urachal carcinoma for more than 15 years. Oncol Lett.

[17] Siefker-Radtke A, Gee J, Shen Y et al (2003) Multimodality management of urachal carcinoma: the M. D. Anderson Cancer Center Experience. J Urol 169(4):1295–1298 10.1097/01.ju.0000054646.49381.01 12629346

[18] Ashley R, Inman B, Sebo T (2006). Urachal carcinoma: clinicopathologic features and long-term outcomes of an aggressive malignancy. Cancer.

